# Incidence and seasonality of Kawasaki disease in children in the Philippines, and its association with ambient air temperature

**DOI:** 10.3389/fped.2024.1358638

**Published:** 2024-04-22

**Authors:** Aden Kay Celis-Seposo, Lina Madaniyazi, Xerxes Seposo, Masahiro Hashizume, Lay Myint Yoshida, Michiko Toizumi

**Affiliations:** ^1^ School of Tropical Medicine and Global Health, Nagasaki University, Nagasaki, Japan; ^2^ Japan Environment and Children's Study Programme Office, National Institute for Environmental Studies, Tsukuba, Japan; ^3^ Department of Hygiene, Graduate School of Medicine, Hokkaido University, Hokkaido, Japan; ^4^ Ateneo Center for Research and Innovation, Ateneo School of Medicine and Public Health, Ateneo de Manila University, Pasig, Philippines; ^5^ Department of Global Health Policy, Graduate School of Medicine, The University of Tokyo, Tokyo, Japan; ^6^ Department of Pediatric Infectious Diseases, Institute of Tropical Medicine, Nagasaki University, Nagasaki, Japan

**Keywords:** Kawasaki disease, seasonality, temperature, environmental epidemiology, children’s environmental health, Philippines

## Abstract

**Background:**

Despite an unknown cause, Kawasaki disease (KD) is currently the primary leading cause of acquired heart disease in developed countries in children and has been increasing in recent years. Research efforts have explored environmental factors related to KD, but they are still unclear especially in the tropics. We aimed to describe the incidence of KD in children, assess its seasonality, and determine its association with ambient air temperature in the National Capital Region (NCR), Philippines from January 2009 to December 2019.

**Methods:**

Monthly number of KD cases from the Philippine Pediatric Society (PPS) disease registry was collected to determine the incidence of KD. A generalized linear model (GLM) with quasi-Poisson regression was utilized to assess the seasonality of KD and determine its association with ambient air temperature after adjusting for the relevant confounders.

**Results:**

The majority of KD cases (68.52%) occurred in children less than five years old, with incidence rates ranging from 14.98 to 23.20 cases per 100,000 population, and a male-to-female ratio of 1.43:1. Seasonal variation followed a unimodal shape with a rate ratio of 1.13 from the average, peaking in March and reaching the lowest in September. After adjusting for seasonality and long-term trend, every one-degree Celsius increase in the monthly mean temperature significantly increased the risk of developing KD by 8.28% (95% CI: 2.12%, 14.80%). Season-specific analysis revealed a positive association during the dry season (RR: 1.06, 95% CI: 1.01, 1.11), whereas no evidence of association was found during the wet season (RR: 1.10, 95% CI: 0.95, 1.27).

**Conclusion:**

We have presented the incidence of KD in the Philippines which is relatively varied from its neighboring countries. The unimodal seasonality of KD and its linear association with temperature, independent of season and secular trend, especially during dry season, may provide insights into its etiology and may support enhanced KD detection efforts in the country.

## Introduction

Kawasaki disease (KD), an acute and self-limited febrile illness of childhood, stands as the foremost cause of acquired heart disease in children in developed countries (1–3). Despite being recognized over 50 years ago, the precise causative agent of KD remains elusive (4). Genetic susceptibility, infection, and immune damage, are considered important factors in the pathogenesis of the disease (5). Notably, children of Filipino descent are more likely to experience aneurysms in KD and are reported to have the highest recurrence rates among non-Asians and non-Filipino Asian communities (6, 7). However, further studies are needed to elucidate the genetic and immunologic factors influencing these findings in this population (6). Recent research efforts have intensified, focusing on infectious triggers and environmental stressors that may potentially activate an immune response in genetically susceptible children (8, 9).

Kawasaki disease is known to exhibit distinct seasonal patterns: higher incidence during the winter season in certain locations, while in others, it peaks during the summer season (10, 11). Although the incidence rates of KD in tropical countries are generally lower compared to Japan, South Korea, and Taiwan, there has been an upward trend in recent years, at least before the Coronavirus Disease 2019 (COVID-19) pandemic in 2020 (10, 12–14). Ambient air temperature, a significant determinant of various health outcomes, has been associated with adverse health effects and mortality (15, 16). Although a few studies have explored the relationship between temperature and KD, the findings have been inconsistent (17–19). One of the limitations of previous research on KD seasonality and its association with temperature is the predominant focus on developed countries like Japan and South Korea, with limited evidence from tropical regions such as the Philippines (10, 11). Moreover, due to distinct climate and population characteristics, findings from other regions cannot be directly extrapolated to tropical countries (11). Therefore, in this study, we aim to describe the epidemiology of KD, to assess its seasonality, and to estimate the association between temperature and KD in the Philippines.

## Materials and methods

This was an ecological study that utilized readily available secondary data obtained from the Philippine Pediatric Society (PPS) disease registry website and National Oceanic and Atmospheric Administration (NOAA) website for KD cases in the National Capital Region, Philippines, and ambient air temperature data, respectively, from January 2009 to December 2019.

Monthly number of KD cases by age group and sex among children 0–18 years old was collected from the PPS disease registry using the International Classification of Diseases-version 10 (ICD-10) code, M30.3 [Mucocutaneous lymph node syndrome (Kawasaki)] (20). KD was set as primary discharge diagnosis under the NCR chapter from January 1, 2009 to December 31, 2019. The PPS disease registry has been an established disease registry by the PPS since May 2006, and is composed of 110 Philippine Pediatric Society-Hospital Accreditation Board (PPS-HAB) accredited pediatric residency training hospitals, as of 2020. It has been the repository of hospital ward discharges from these pediatric training hospitals across the country and has served as the main reference of these hospitals for the reported childhood diseases (21, 22). This study also utilized population data during the period of 2009 to 2019 from the Philippine Statistics Authority (PSA) (23).

Ambient air temperature (hereafter temperature) data was obtained from the Global Summary of the Day (GSOD), Version 1 dataset of NOAA (24). The daily temperature dataset used came from the three background monitoring stations in NCR, i.e., Manila Port, Ninoy Aquino International Airport (NAIA), and Science Garden (Supplementary Figure S1). Daily mean temperature data were averaged across the three stations and were converted from degrees Fahrenheit to degrees Celsius. Then it was aggregated to a monthly scale to match the temporal scale of the outcome of interest. Time series plot of monthly number of KD and monthly mean ambient temperature was generated to show possible association graphically (Supplementary Figure S2).

### Statistical analyses

To determine the incidence of KD in children, we utilized the annual number of KD cases and the population of children for different age groups, i.e., 0–4, 5–9, 10–14 and 15–18 years old, as the numerator and denominator, respectively. Since the available annual population data from PSA were 2010, 2015, 2016, 2017, 2018 and 2019 only, we calculated the annual population in 2009 and 2011–2014 using linear interpolation (25, 26). Next, we used a generalized linear model (GLM) with Poisson regression accounting for overdispersion (via quasi-likelihood estimation) to assess the seasonality of KD and estimate its association with temperature (27, 28).

#### Assessing seasonality of Kawasaki disease

We used a pair of sine and cosine functions to assess seasonality of KD in annual cycle based on monthly data (27) using Equation 1:

(1) $$\;\;\;\;\;\;\;\;\;\;\;\;\begin{array}{rl} & \;Y_{t}∼\mathrm{Quasi}\text{-}\mathrm{poisson}(E(Y_{t}))\\ & \log(E(Y_{t}))=\alpha +c\;\cos(\omega_{t})+s\;\sin(\omega_{t})+\beta \;\mathrm{Year}+\varepsilon ,t=1,\ldots ,12, \end{array}$$

where $Y_{t}$ was the monthly KD cases on time *t* following a quasi-Poisson distribution; α was the intercept; $\omega_{t}$ was the radians transformed from $2\pi f_{t}$, where $f_{t}=(\mathrm{mont}h_{t}-1)/12$, since we were estimating the annual cycle using monthly data; *c* and *s* were the coefficients derived from $c\;\cos(\omega_{t})+s\;\sin(\omega_{t})$, respectively; Year was a linear function controlling for the long-term trend; ε was the error term. We summarized the seasonality of KD from its shape, timings (i.e., peak and trough), and size: (a) we used sine and cosine coefficients to derive predicted seasonal shape, (b) the month with maximum and minimum estimates of KD cases were identified as peak and trough, respectively, and (c) the difference in the maximum estimates of KD cases and the minimum estimates of KD cases was calculated to measure the amplitude (both in relative and absolute scale) (29).

#### Assessing association between temperature and KD

Then, we included a linear function for temperature in the model above to estimate the association between temperature and KD:

(2) $$\;\;\;\;\;\;\;\;\;\;\;\;\;\;\begin{array}{rl} & \;Y_{t}∼\mathrm{Quasi}\text{-}\mathrm{poisson}\;(E(Y_{t}))\\ & \log(E(Y_{t}))=\alpha +\beta_{1}\mathrm{Temp}+c\;\cos(\omega_{t})+s\;\sin(\omega_{t})+\beta \;\mathrm{Year}+\varepsilon \end{array}$$

where Temp was a linear function for main exposure variable: temperature. We further restricted the analysis to dry (June to November) and wet seasons (December to May) and examined the association in two seasons separately by using the same model in Equation 2.

To examine the potential nonlinearity of the temperature-KD association, we utilized natural cubic splines for the temperature with varying degrees of freedom (df=2,3,4,5) and compared the linear and nonlinear parameters for temperature by using Quasi Akaike's Information Criterion (QAIC).

All statistical analyses were performed with R (version 4.1.1) using season and dplyr packages (R core team, 2021). The 2-sided statistical tests were considered significant with *p*-value less than 0.05.

Ethical approval was obtained from the Ethics Committee of Nagasaki University School of Tropical Medicine and Global Health (IRB Approval No. 202).

## Results

**Figure 1:**
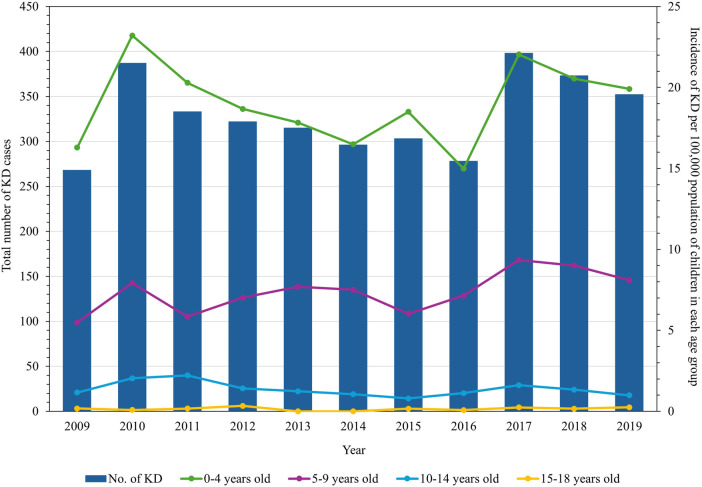
Annual number of Kawasaki disease cases in children in the National Capital Region, Philippines, and its incidence rates per age group from 2009 through 2019.

A total of 3,625 KD cases from NCR were recorded in the period of January 2009 to December 2019. Among these, 2,484 (68.53%) cases belonged to the age group of zero to four years old, with 2,134 (58.87%) cases being males. The male:female ratio was 1.43:1. The monthly mean temperature in NCR during the study period was 28.08°C, with the wet season having a higher mean temperature than the dry season (Table 1). The annual incidence of KD during the study period was 14.98–23.20 cases per 100,000 population of children less than 5 years old (Figure 1). The incidence of KD among other age groups was comparatively lower (Supplementary Table S1).

**Table 1:** Summary statistics of monthly number of Kawasaki disease cases in children and ambient air temperature in the National Capital Region, Philippines, from 2009 to 2019.

	*N* (%)	Monthly number of KD cases					
		Mean	Min	25th	50th	75th	Max
Total	3,625 (100.00)	27.46	10.00	20.00	27.50	33.25	51.00
Sex							
Male	2,134 (58.87)	16.17	5.00	11.75	16.00	20.00	34.00
Female	1,491 (41.13)	11.30	0.00	8.00	11.00	14.25	21.00
Age group (in years)							
0–4	2,484 (68.52)	18.82	7.00	14.00	18.00	23.00	37.00
5–9	949 (26.18)	7.19	1.00	4.00	7.00	9.00	20.00
10–14	172 (4.74)	1.30	0.00	0.00	1.00	2.00	7.00
15–18	20 (0.55)	0.15	0.00	0.00	0.00	0.00	2.00
		Temperature (°C)					
		Mean	Min	25th	50th	75th	Max
All-year round	–	28.08	25.15	27.45	27.96	28.63	31.12
Season-specific							
Dry season^a^	–	28.02	25.15	26.84	27.68	29.37	31.12
Wet season^a^	–	28.14	27.02	27.69	28.04	28.39	30.01

^a^ Dry season, December to May; Wet season, June to November.

**Figure 2:**
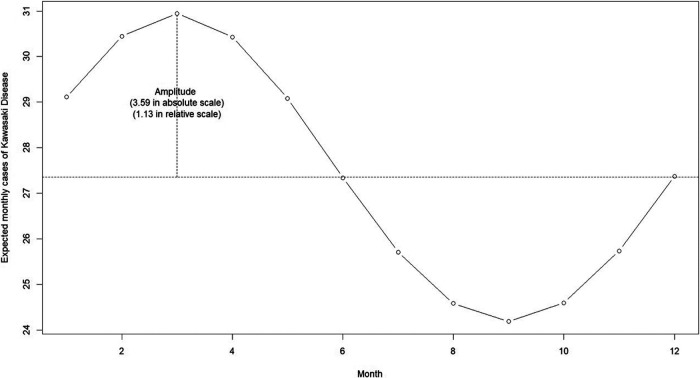
Estimated annual seasonal pattern of Kawasaki disease cases from the National Capital Region, Philippines.

Figure 2 showed the estimated annual seasonal pattern of KD cases. We found that the seasonal pattern of KD displayed a unimodal shape with a marked increase of cases at the onset of the dry season (from December to May). The highest number of cases was reported in March (i.e., peak), with cases declining during the wet season (from June to November), reaching its lowest in the month of September (i.e., trough). The average number of cases was 27.35 cases per month. The amplitude was at 1.13 in relative scale which meant that rate ratio of KD cases was 1.13 times higher than the average in March; and 1.13 times lower than the average in September.

Table 2 illustrated the linear association between temperature and KD. The result showed a statistically significant positive association between the two, with an 8.28% (95% CI: 2.12%, 14.80%) increase in monthly KD cases for every one-degree Celsius rise in the monthly mean temperature, after adjusting for seasonality and long-term trend. Similarly, a positive association was observed during the dry season, with a 6.0% (95% CI: 1.0%–11.0%) increase in monthly KD cases for every one-degree Celsius increase in monthly mean temperature. Whereas during the wet season, there was insufficient evidence to show an association (RR = 1.10, 95% CI: 0.95–1.27). Finally, we explored the potential nonlinearity of temperature-KD association and found that the linear model provided the best fit (Supplementary Table S2).

**Table 2:** Estimates from the linear association of ambient air temperature and Kawasaki disease cases from the National Capital Region, Philippines using quasi-poisson regression.

Temperature	RR (95% CI)^b^	*p*-value
All-year round	1.08 (1.02–1.15)	**0** **.** **01**
Season-specific		
Dry season^a^	1.06 (1.01–1.11)	**0** **.** **01**
Wet season^a^	1.10 (0.95–1.27)	0.22

Bold values indicate significantly associated estimates.

^a^ Dry season, December to May; Wet season, June to November.

^b^ RR, relative risk; 95% CI, 95% confidence interval.

## Discussion

Our study described the annual KD incidence in the Philippines which showed variability when compared to other countries worldwide. Additionally, we found that KD cases exhibit unimodal seasonality with its peak occurring in March and the trough in September. The effect of year-round ambient air temperature was linearly associated with monthly number of KD cases, with noticeable effect particularly during the dry season of December to May. However, no such association was observed during the wet season. To the best of our knowledge, this is the first population-based study in the Philippines to investigate the seasonality of KD and its association with temperature.

Similar to studies previously conducted (9, 30, 31), majority of KD in children in the Philippines belong to less than five years old age group. One major factor that the scientific community sees as contributing to the higher incidence of KD in younger age groups is the predominance of innate immunity in these age groups and subsequent inflammatory responses linked to the disease (32, 33). Activation of the innate immune system, particularly the endothelial Nlrp3 inflammasome induced by certain triggers, may lead to the development of coronary arteritis, which is a key feature in KD vasculitis (32, 34). Some studies also underscore the increased exposure of these children to several infectious agents, supporting a possible infectious etiology of the disease (35). A review of the hygiene hypothesis by Lee (36) has shown that the age group of 6 months to 2 years old has low levels of immunoglobulin G (IgG) due to waning maternal antibody levels and low immune development. This, in turn, increases their risk of developing KD due to poor antibody-mediated B cell immunity. On the other hand, the cause of gender predominance in KD has yet to be elucidated.

This study provides evidence of the incidence of KD in children in the Philippines, which shows substantial variation compared to its neighboring countries. For instance, Thailand reported a lower incidence rate with 2.14 to 3.43 cases per 100,000 children aged 0 to 5 years old from 1998 to 2002 (37), while Malaysia and Singapore showed a relatively similar incidence with 14.8 cases in 2006–2019 and 51.4 cases per 100,000 population of less than 5 years old, respectively, during 2006–2019 and 2021 (38, 39). This difference in the rates of occurrence may be attributed to various methodologies employed, as some countries have estimated the occurrence rates based on available isolated hospital records or existing national surveillance systems (10). While Japan, South Korea, and Taiwan have used robust nationwide data which have contributed significantly to the epidemiological investigations of KD, developing countries, particularly those in Southeast Asia, have limited accurate nationwide data reporting due to the absence of centralized data linkages (10, 40). It is suggested that active nationwide surveys and national health insurance data management be utilized to gain a comprehensive understanding of the origin of KD and provide useful insights for future research (31).

Seasonal clustering of KD has been widely explored for its potential to elucidate the etiology of the disease; however, the results are inconsistent. A comprehensive review by Burns et al. (11) on KD seasonality across the globe has highlighted no distinguishable seasonal variation in the tropics and Southern Hemisphere extratropics primarily due to scarcity of time-series studies and diverse climates across the study locations. Our study showed increasing KD cases at the onset of the dry season, peaking in March, and reaching its nadir in September. This pattern mirrors the incidence observed in several countries. For example, in Taiwan, the peak season is from April to June, while in Japan, it is from January to March (10, 11, 41). In Mainland China, the peak incidence occurs during spring (March to May) and summer (June to August) (42). The disease is most prevalent in San Diego in late winter and early spring (January to March) (43). The Philippines, characterized as a tropical country, experiences two distinct seasons: the wet season, which spans from May to November, and the dry season, from December to April, which can be further divided into the cool, dry season (December to February), and the hot, dry season (March to April) (44). During the latter season, the temperatures are mild and increase sharply towards the end of April or the beginning of May, with relatively low humidity, providing a more conducive climate for outdoor activities (44, 45). The peak incidence of KD in the aforementioned countries may be attributed to the cool temperatures and low relative humidity during these months. Compared with those studies conducted in locations with similar climates; however, our result is different. For instance, a study conducted in Malaysia revealed that the highest number of KD cases were reported during the rainy season, from October to December (38). In Singapore, KD cases peaked in April, with the lowest number between February and March, coinciding with frequent rainfall during this season (46). These disparities in seasonality indicate that not only climate, but also other factors such as large-scale tropospheric wind patterns, environmental exposures, and host genetics, can influence the seasonality of KD (11). Specifically, the seasonal patterns of KD are influenced by airborne triggers carried by wind currents from central Asia (47), exposure to infectious agents or allergens in the environment leading to KD occurrence based on the child's genetic background and susceptibility (9, 10, 48), variations in infectious disease activity in each season (49, 50), dynamic population activity exposing the vulnerable to the infectious agents in the environment (8), and effect of meteorological factors such as large-scale tropospheric wind patterns and ambient temperature (11, 47, 51). Nonetheless, these theories are yet to be directly confirmed in several studies.

Seasonal patterns of infectious diseases are also influenced by several factors such as meteorological conditions, host-pathogen interactions, and human behavior (52). Studies conducted in the Philippines have shown that some infectious diseases display discernible seasonality. However, the present study has found no clear correlation between the dry season peak of KD and seasonality of specific infectious diseases in the country. Notably, leptospirosis, dengue fever, and infectious diarrhea usually occur during the rainy season from July to November, a period that provides ideal conditions for pathogen transmission (53, 54). In another study, community-acquired pneumonia incidence in infants reaches its seasonal peak after a period of undernutrition also typically during the rainy season (55). Conversely, the respiratory syncytial virus presents a robust seasonal peak during the cold and dry months of January and February in the country (56). Nevertheless, further studies are needed to elucidate a clear relationship between these infectious agents and KD. While there are a few studies on KD seasonality and its etiology in the tropical region, the results of this study could add to the existing evidence of seasonal variation from a tropical country.

Studies related to the association between temperature and KD incidence are sparse and demonstrate considerable variation across countries worldwide (17, 19, 50). Our findings have provided compelling evidence that suggests that as temperature increases, the risk of occurrence of KD also increases. Several mechanisms have been postulated to explain this increased risk of developing KD in higher temperature (50, 57). These include enhanced spread of infectious agents such as fungi and bacteria in high ambient temperature, increased exposure to infectious agents due to children spending more time outside during summertime, efficient release of inflammatory mediators upon exposure to high temperature causing systemic inflammatory response and vascular endothelial dysfunction, and increased incubation of infectious agents in the environment during hot weather (8, 50, 57, 58). In contrast, studies conducted in Japan, San Diego, and Michigan have shown that there is an inverse relationship between daily average temperature and the number of admissions for KD (17, 19, 59, 60). While there are limited studies on temperature and seasonality in KD, the contrasting evidence, probably due to varied methodologies, underscores the complexity of its etiology. Furthermore, the impact of short-term variations in other meteorologic conditions such as relative humidity, wind patterns, and amount of rainfall on KD occurrence remain inconclusive (17, 19, 61, 62). Therefore, further research is needed to look into the interaction of these meteorological factors, microbial infection, and environmental triggers on KD occurrence.

Our study has several strengths and limitations that warrant attention. First, its novelty is highlighted as this is the first epidemiological study of KD conducted in the Philippines. Second, the investigation on the seasonality and temperature association with KD in a tropical country is also a significant contribution to the existing knowledge gap. However, it is important to note several limitations which include the use of administrative health data in this study that had limited health categories since the country has no centralized reporting system for KD. This could have led to an under or overestimation of the number of KD cases throughout the study period. Also, the current data from the registry only represent cases from the NCR and may not represent the whole population in the Philippines. Moreover, individual-level exposure to temperature could not be measured or estimated as it is an ecological study, hence the result of this study should be carefully interpreted. Finally, due to limited availability of health data from the registry, the use of monthly data may have restricted us from exploring the potential delayed effects of temperature on KD.

## Conclusion

In conclusion, we have presented the annual incidence of KD in NCR, Philippines and highlighted its variation with its neighboring countries attributable to differences in methodologies employed. The occurrence of KD showed a unimodal seasonal variation with a linear association to temperature, especially during the dry season from December to May. These findings may aid ongoing investigations regarding the etiology of KD and the environmental factors that influence its occurrence. Additionally, the results of this study may improve disease recognition in the Philippines, leading to timely management and prevention of long-term complications.

## Data Availability

The datasets presented in this study can be found in online repositories. The names of the repository/repositories and accession number(s) can be found below: https://pps.ivant.com/search.do, https://www.ncei.noaa.gov/metadata/geoportal/rest/metadata/item/gov.noaa.ncdc%3AC00516/html#.
