# Supplementary material for: Incidence and seasonality of Kawasaki disease in children in the Philippines, and its association with ambient air temperature
Source: Front Pediatr. 2024 Apr 22;12:1358638. doi: 10.3389/fped.2024.1358638 (PMC11070490; doi:10.3389/fped.2024.1358638)
Supplement: Supplementary file 1 [file Table1.pdf]

Supplementary Table S1. Total annual number of Kawasaki disease cases in each age group with its computed incidence per 100,000 population of children per age group from NCR, Philippines, 2009 to 2019.

Year	0-4 years old		5-9 years old		10-14 years old		15-18 years old	
	**N**	**Incidence**	**N**	**Incidence**	**N**	**Incidence**	**N**	**Incidence**
2009	190	16.29	63	5.49	13	1.16	2	0.17
2010	272	23.20	91	7.92	23	2.04	1	0.09
2011	239	20.29	67	5.84	25	2.22	2	0.17
2012	221	18.67	81	7.02	16	1.42	4	0.33
2013	212	17.83	89	7.69	14	1.24	0	0.00
2014	197	16.49	87	7.50	12	1.06	0	0.00
2015	222	18.49	70	6.02	9	0.79	2	0.16
2016	180	14.98	84	7.15	13	1.13	1	0.08
2017	265	22.04	111	9.34	19	1.62	3	0.24
2018	247	20.55	108	9.01	16	1.34	2	0.16
2019	239	19.91	98	8.10	12	0.99	3	0.25
