# Supplementary material for: Incidence and seasonality of Kawasaki disease in children in the Philippines, and its association with ambient air temperature
Source: Front Pediatr. 2024 Apr 22;12:1358638. doi: 10.3389/fped.2024.1358638 (PMC11070490; doi:10.3389/fped.2024.1358638)
Supplement: Supplementary file 2 [file Table2.pdf]

Supplementary Table S2. Quasi Akaike's Information Criterion generated from the different model analyses.

Model	QAIC
Linear model using GLM	994.0571*
Nonlinear model using NS:	
df 2	1021.020
df 3	1025.159
df 4	1029.869
df 5	1034.014

Note: The linear model* for temperature-KD association showed the best fit.
QAIC: Quasi Akaike's Information Criterion
GLM: generalized linear model
NS: natural cubic spline
df: degrees of freedom
