# Supplementary material for: Incidence and seasonality of Kawasaki disease in children in the Philippines, and its association with ambient air temperature
Source: Front Pediatr. 2024 Apr 22;12:1358638. doi: 10.3389/fped.2024.1358638 (PMC11070490; doi:10.3389/fped.2024.1358638)
Supplement: Supplementary file 3 [file Image1.pdf]

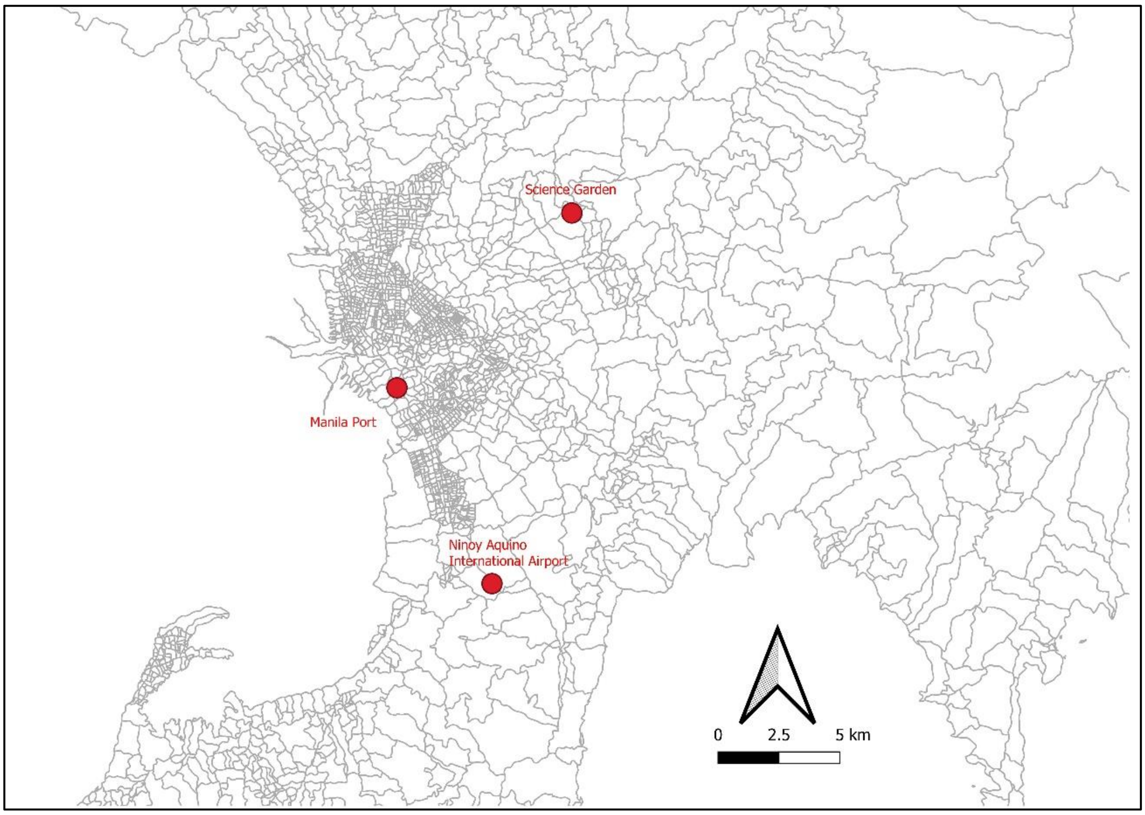

Supplementary Figure S1. The three background monitoring stations located in NCR where temperature dataset was obtained.
