# Supplementary material for: Incidence and seasonality of Kawasaki disease in children in the Philippines, and its association with ambient air temperature
Source: Front Pediatr. 2024 Apr 22;12:1358638. doi: 10.3389/fped.2024.1358638 (PMC11070490; doi:10.3389/fped.2024.1358638)
Supplement: Supplementary file 4 [file Image2.pdf]

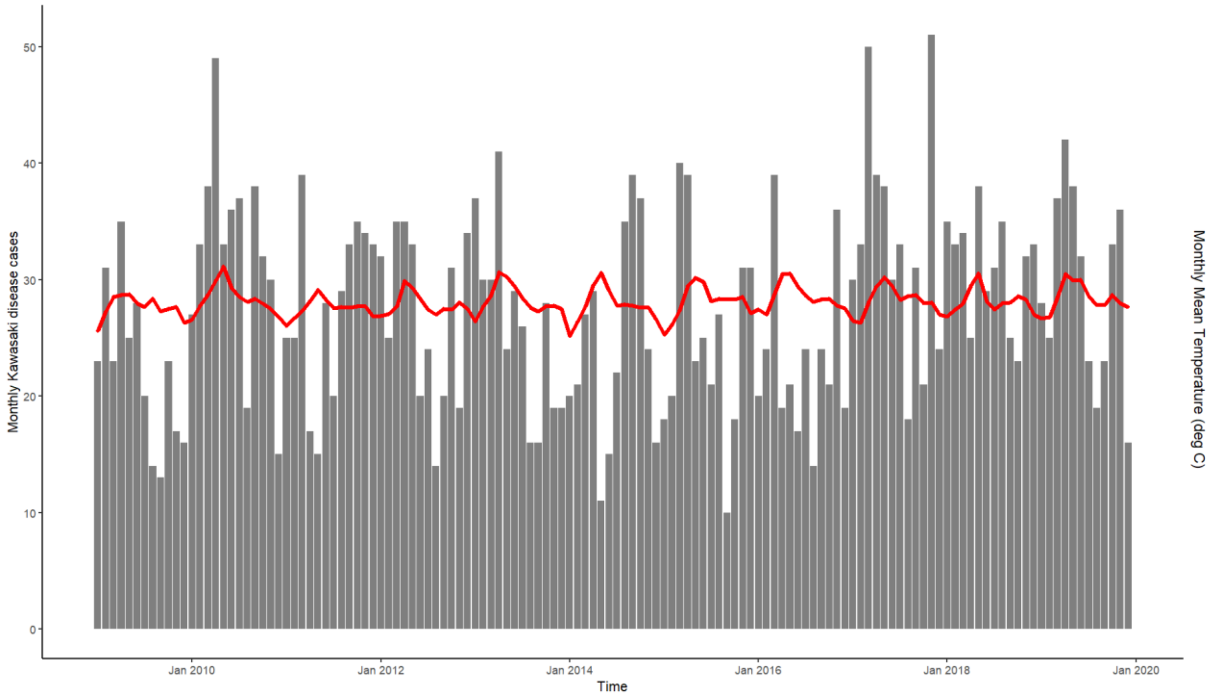

Supplementary Figure S2. Time series plots of monthly number of KD (A) and monthly mean ambient temperature (in degree C) (B) from January 2009 to December 2019 in NCR.
